# Orbital Subperiosteal Hematoma Arising in the Context of Chronic Sinusitis: Suspicion of Association

**DOI:** 10.5334/jbsr.1829

**Published:** 2019-07-03

**Authors:** Kerwin Roelandt, Laurence Van Ruyssevelt, Pierre Bosschaert

**Affiliations:** 1 Clinique Saint-Pierre, BE; 2 Clinique Saint-Pierre Ottignies, BE

**Keywords:** subperiosteal, orbital, hematoma, non-traumatic, sinusitis

A 76-year-old woman who experienced sinusitis for years was presented to the emergency department complaining of left orbital pain, eyelid swelling, and reduced visual acuity. She was nonfebrile, and she reported no recent trauma nor any violent Valsalva maneuver.

Ophthalmological examination showed a proptosis and a limitation of eye movements, particularly in the up gaze (Figure 1) as well as a loss of visual acuity of 2/20. Visual field was not initially evaluable. Intraocular pressure remained within normal limits. Blood analysis showed moderate inflammation and ruled out coagulative disorders.

**Figure 1:**
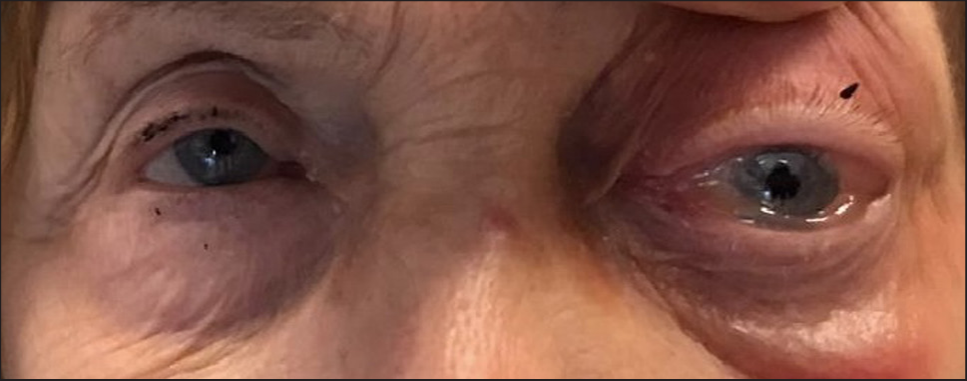


Non-contrast computed tomography (NCCT) revealed a biconvex well-defined 41 × 31 × 17 mm collection with layering hyperdense blood products in the roof of the orbit, displacing superior oblique and rectus muscles, causing exophthalmia and deformity of the eye. No obvious bone erosion was visualized (Figure 2, asterisk).

**Figure 2:**
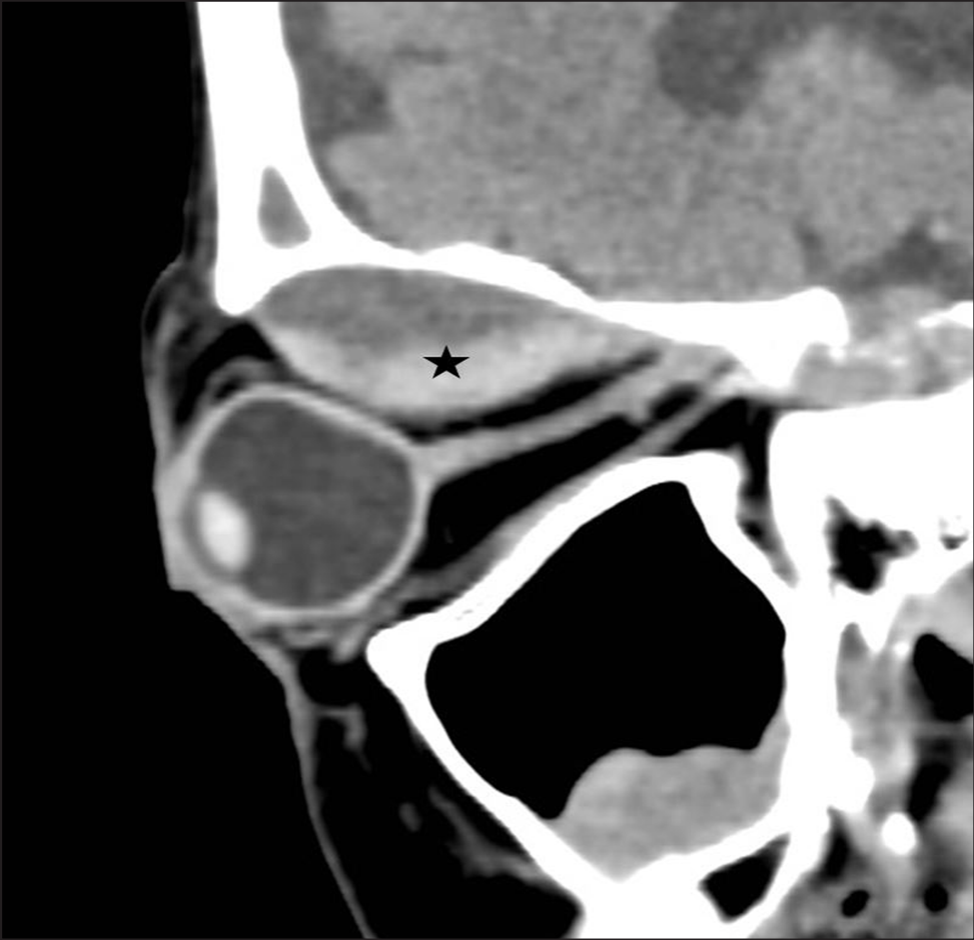


Magnetic resonance imaging (MRI) indicated an acute to subacute hematoma with hypointense signal on T2/FLAIR-weighted images (Figure 3a and **b**, arrows) and isointense to hyperintense signal on T1-weighted images (Figure 3c). Diffusion-weighted imaging was perturbated, as usual in a hematoma (Figure 3d). A subtle peripheral enhancement was noted after Gadolinium administration, probably caused by a reactional inflammation. MRI also confirmed the absence of infiltration of the retro-orbital fat and excluded a cerebral venous sinus thrombosis. Optic nerve was normal.

**Figure 3:**
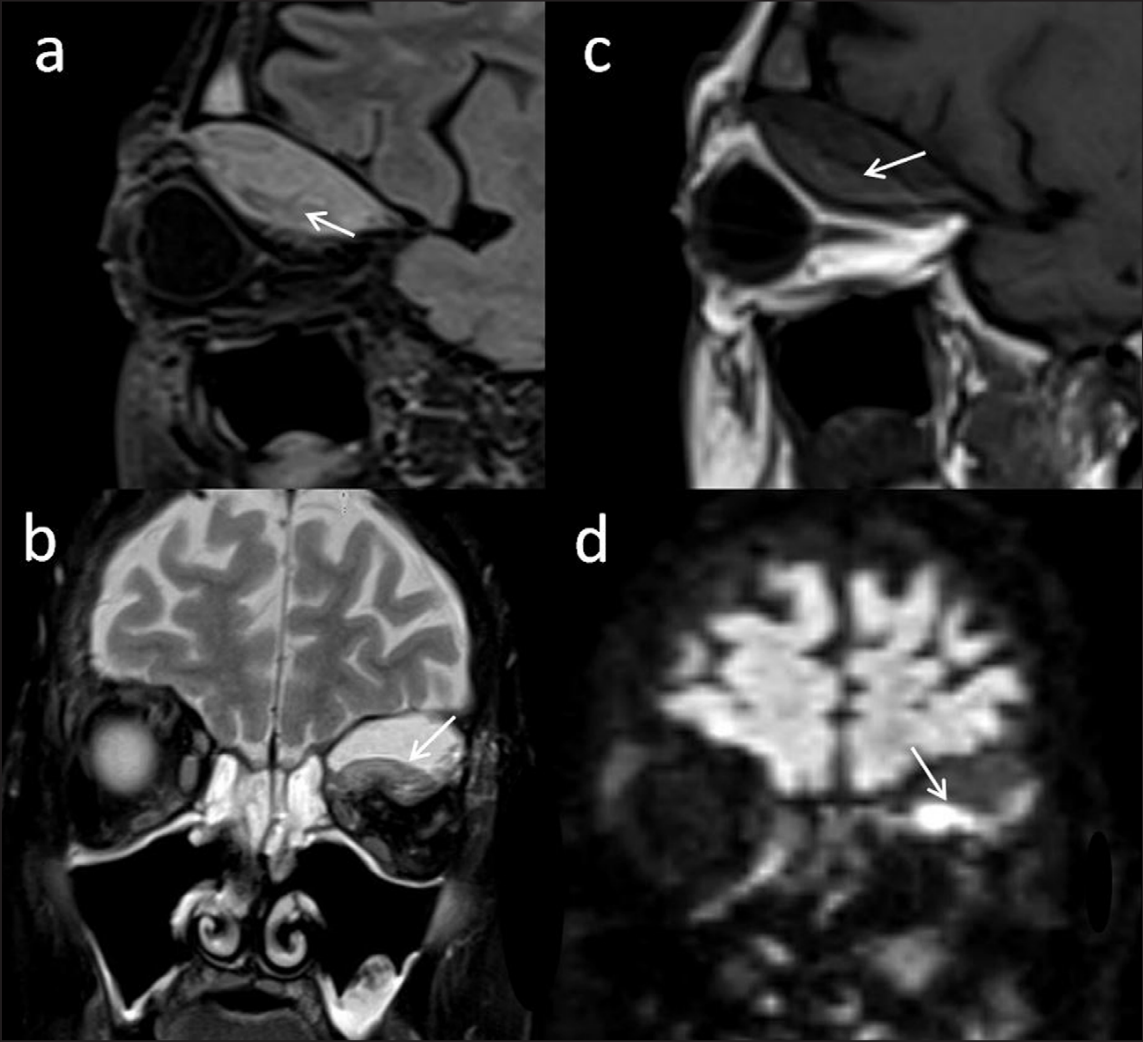


The patient underwent a surgical drainage of the subperiosteal space proving the presence of blood, but no microorganisms were demonstrated.

The postoperative clinical follow-up showed progressive recovery of muscle motricity and the return to normal of visual acuity (20/20). The visual field showed an altitudinal inferior defect with a macular sparing.

Orbital MRI control performed one month postoperatively was unremarkable.

## Comment

Most cases of orbital subperiosteal hematoma (OSH) typically result from the rupture of blood vessels in the setting of trauma. Non-traumatic OSH is uncommon and typically associated with a sudden increase in cranial venous pressure, as in emesis, bleeding diathesis or chronic sinusitis [1]. OSH arising in the context of chronic sinusitis is considered by many authors as an extremely rare complication, with few more than 20 cases reported in the literature. In some patients, it may be impossible to assign an underlying cause to the OSH. In our case, no bone defect is visualized. The age range is broad, children and elders being more frequently affected, with a female preponderance. The superior orbit is the most common location of OSH, regardless of the site of sinus involvement.

Because the possibility of orbital infection, surgical drainage is recommended. An anterior orbital approach is preferred. Early detections and treatment are necessary to prevent compressive optic neuropathy and to ensure the best prognosis. Antibiotic coverage is recommended.

While computed tomography performs well to show sinusitis, possible bone changes, and the presence of blood collection or abscess, an MRI is superior for evaluating intraorbital structures, especially the optic nerve.
